# Health-related quality of life of ambulant adults with cerebral palsy and its association with falls and mobility decline: a preliminary cross sectional study

**DOI:** 10.1186/s12955-014-0132-1

**Published:** 2014-08-30

**Authors:** Prue E Morgan, Sze-Ee Soh, Jennifer L McGinley

**Affiliations:** Physiotherapy Department, School of Primary Health Care, Monash University, PO Box 527, Frankston, Victoria Australia; Physiotherapy Department, Caulfield Hospital, Caulfield, Australia; School of Physiotherapy, Melbourne School of Health Sciences, The University of Melbourne, Melbourne, Australia

## Abstract

**Background:**

Despite an increasing number of studies examining the profile of falls and mobility decline in adults with cerebral palsy (CP), little is known about its impact on an individual’s life quality. The aim of this preliminary study was to assess the wellbeing and health status aspects of health-related quality of life (HRQOL) in ambulant adults with CP and explore the relationship of falls and mobility decline with HRQOL.

**Method:**

Ambulant adults with CP completed postal surveys which sought demographic data, mobility (Gross Motor Function Classification System; GMFCS-E&R), presence of mobility decline, falls history, and HRQOL (Personal Wellbeing Index (PWI), Short Form-36 Health Survey (SF-36)).

**Results:**

Thirty-four community-dwelling ambulant adults with CP with a mean age of 44.2 years (SD; 8.6; range 26–65) participated. Twenty-eight (82%) participants reported mobility decline since reaching adulthood, and a similar proportion of individuals (82%) reported having had more than two falls in the previous year. The health status and wellbeing of this sample of ambulant adults with CP were generally lower compared with the Australian normative population. Mobility decline was found by univariate regression analysis to be associated with mental health status (β = 0.52; *p* = 0.002), but not when other predictor variables were included in the multivariate model (β = 0.27; *p* = 0.072). In contrast, self-reported history of falls was found to be a significant contributing factor for both physical health status (β = −0.55; *p* = 0.002) and personal wellbeing (β = −0.43; *p* = 0.006).

**Conclusions:**

This sample of ambulant adults with CP perceived their HRQOL to be poor, with some health status and wellbeing domains below that of population wide comparisons. A majority of these individuals also experienced a fall in the last year and a decline in their mobility since reaching adulthood. While further research is required, this preliminary study has highlighted the potential implications of falls and mobility decline on HRQOL in adults with CP.

## Background

Quality of life is a critical factor to consider when seeking an understanding of the experiences of individuals ageing with a disability. Quality of life (QOL) is defined by the World Health Organisation as an “individual’s perception of their position in life in the context of the culture and value systems in which they live and in relation to their goals, expectations, standards and concerns” (p.1405) [1]. It is a multi-dimensional construct that includes aspects of health-related QOL (HRQOL), social relationships, personal characteristics, global issues and socioeconomic status [2]. The health dimension of QOL is of particular relevance to individuals with disability, particularly adults with cerebral palsy (CP), as it takes into account aspects of physical health, emotional status and cognitive ability [3]. A number of health conditions and functional deteriorations have been described by adults ageing with CP [4-6], as a direct or indirect consequence of their original diagnosis which have the potential to impact on HRQOL.

Of particular concern, is the loss of or decline in independent mobility for adults with CP, reportedly experienced by around 25-40% of ambulant adults [7,8]. Maintaining the ability to walk efficiently and safely is desirable for adults with CP to enable and enhance social participation, and retain independence. Increasing problems with falls and balance are frequently cited as both causes and potential consequences of mobility decline in this population [9,10]. Recent studies have begun to explore the profile and physical sequelae of falls in adults with CP [11,12], however the impact of falls and near-falls and mobility decline on HRQOL is not yet established.

Functional mobility has been associated with HRQOL across a range of acquired health conditions in adults such as stroke [13], multiple sclerosis [14], and Parkinson’s disease [15], with poorer performance linked with poorer HRQOL. The relationship between mobility status and decline, and HRQOL is less clear in CP. Livingston summarised a number of studies of adolescents with CP reporting that mobility status was associated with physical, but not necessarily psychosocial wellbeing [16]. However, Tarsuslu and colleagues were unable to find any relationship between mobility status and measures of HRQOL in their cohort of young adults (mean age 28 years) with CP [17]. It is recognised that secondary impairments such as mobility decline most commonly arise in the mid-thirties to forties of ambulant adults with CP [8], possibly limiting extrapolation of Tarsuslu and Livingston’s findings to this age group. In older, predominantly ambulant adults with CP (mean age ≥40 years), associations of physical aspects of HRQOL with pain [18-20], and fatigue [21] have been established. Furthermore, an Australian study demonstrated an association between total physical activity and the physical subscales of a HRQOL measure in adults with CP (mean age 38 years, 54% non-ambulant) [22]. We do not know however whether there is a relationship between the presence of mobility decline and subsequent HRQOL in ambulant adults with CP.

For older adults without disability, it is well recognised that a fall or near-fall may be a key event that triggers a deteriorating illness trajectory, increased need and dependency and resultant decline in QOL [23]. For those following stroke, falls may be associated with depressive symptomatology [24] and in individuals with Parkinson’s disease self reported falls history has a direct relationship with HRQOL [15]. Older adults and those with acquired neurological dysfunction may reduce their activity levels as a result of falls or near-falls, often due to the development of psychological sequelae such as fear of falling [25,26], further adversely affecting HRQOL. In adults with CP, little is known about the relationship between falls and QOL. Hirsh and colleagues [6] found no significant relationship between psychological function (as measured by the mental health components of the Short-Form 36 Health Survey, [27]) and reports of ‘imbalance’ in a mixed group of ambulant and non ambulant adults with CP. The relationship between falls, mobility decline and measures of HRQOL in adults with CP warrants further exploration.

The aims of this study were to describe and quantify the health status and wellbeing aspects of HRQOL in a sample of ambulant Australian adults with CP, and to assess the relationships between HRQOL and the presence of falls and mobility decline.

## Method

### Participants

Community dwelling ambulant adults with CP of any subtype [28], aged 18–65 years and Gross Motor Function Classification System – Extended and Revised (GMFCS-E&R) Level I-III [29] were invited to participate through advertisements placed at physiotherapy clinics, health facilities and community agencies such as adult community activity groups and disability-specific organisations. Interested participants were invited to contact the researcher for a short interview to clarify inclusion criterion. Self-report of a diagnosis of cerebral palsy made by a medical practitioner was accepted. Participants were excluded if English language ability was insufficient, or if cognitive impairment precluded the ability to follow instructions to enable participation using the Abbreviated Mental Test Score (AMTS) [30]. As per Hodkinson’s original findings (Sensitivity = 70-80%, Specificity = 71-90%) [30], participants were excluded if they scored less than 7/10 on the AMTS. Ethical approval was gained from Monash University (MUHREC - LR - 2011001612).

### Procedure

A survey was developed with consumer feedback from three ambulant adults with CP. The feedback was used to guide the language, descriptors and format of the survey by ensuring that the font size and layout was suitable for potential participants who may have limitations of visual acuity and/or fine motor skills. The survey was mailed to participants with a reply paid envelope, or distributed electronically via email if requested. The survey consisted of standardised tools and purpose designed questions, and took approximately 30 minutes to complete. Participants could elect to have a carer assist with survey completion after providing verbal responses to questions.

### Outcome measures

The survey sought information including participant characteristics and demographic data including age, gender, CP subtype [28] and self-reported GMFCS-E&R level [29]. Self-nomination of current GMFCSE&R level has been shown to have excellent agreement with professional ratings [31]. Participants were also asked to report their self-perceived change in mobility since 18 years of age (‘is your walking better, the same or worse than when you were 18 years old?’) [9], and the number of falls they had in the previous 12 months. A lay definition of a fall (‘an unexpected event in which you come to rest on the ground, floor, or lower level’) was provided in the survey to ensure that the falls data obtained was accurate [32]. Participants were classified as repeat fallers if they had more than 2 falls a year and infrequent/non-fallers if they had between 0 and 2 falls a year [33]. Previous studies in older adults have shown that there is a reasonable relationship between retrospective self-report and prospective calendar report of falls with a trend towards under-reporting of falls in retrospective method [34].

Many measures of HRQOL are available and can be categorised as those exploring (i) health utility (the value a person places on their health), (ii) health status (the ability to perform activities of daily living) and (iii) well being (satisfaction with life) [2]. The Short Form-36 Health Survey (SF-36) is an example of a health status instrument that quantifies an individual’s degree of health [27,35], while the Personal Wellbeing Index (PWI) [36]) is an example of a ‘wellbeing’ measure that focuses on how satisfied an individual is with their life. To date, there is no disease-specific tool that can be used to measure the HRQOL of adults with CP. The SF-36 and PWI were used in this study because both tools measure complementary aspects of HRQOL [2] and have Australian normative values available for comparison. As such, the SF-36 and PWI was considered to be culturally and contextually appropriate for the Australian setting.

The SF-36 [27,35] is a self-report questionnaire with 36 items which measure eight dimensions: physical functioning, social functioning, role limitations due to physical problems, role limitations due to emotional problems, mental health, energy and vitality, pain and general perception of health. It yields an 8-scale profile of functional health scores, as well as two summary measures – a physical component summary scale (PCS) and a mental component summary scale (MCS). The scores range from 0 to 100, with higher scores representing better health status. In Australia, age- and gender-matched reference data is available (*n* = 20,000), with the MCS and PCS expected to have a mean of 50 (SD 10) in the general population [37].

Wellbeing was quantified using the PWI [36] where eight questions about satisfaction with life domains are scored on an 11-point scale (0–10). Responses are converted to a percentage of scale maximum [36], with higher scores indicating greater wellbeing. Data is reported as eight individual domains, with domain scores aggregated and averaged to form an overall PWI summary index (PWI-SI) that ranges from 0 to 100. Reference values are available for the Australian population (n = 55,000), with a mean of 75 reported for the PWI-SI [36].

### Data analysis

Descriptive statistics were used to summarise the demographic data and scores derived from the SF-36 and PWI. Non-parametric statistics such as the Friedman test was used to compare the ratings for the dimensions of HRQOL as measured by the SF-36 and PWI with Bonferroni adjustment for multiple comparisons (*p* < 0.002 and *p* < 0.0024 respectively) due to the small sample size and underlying skewed distribution. To compare the health status and wellbeing of ambulant adults with CP and other comparable populations, 95% confidence intervals (CI) were constructed around the estimates. The Mann Whitney U test of significance with Bonferroni adjustment for multiple comparisons was also used to explore differences in SF-36 (*p* < 0.006) and PWI (*p* < 0.007) ratings between individuals who perceived a decline in their mobility or had more than 2 falls in the last 12 months, and those who did not.

Univariate and multivariate linear regression analyses were performed to examine whether mobility decline and self-reported history of falls contributed to aspects of HRQOL of ambulant adults with CP. The dependent outcome variables were mental and physical health status as measured by the MCS and PCS of the SF-36, and wellbeing as measured by the PWI-SI. The independent variables included in the analyses were age, disease severity, self-reported history of falls and mobility decline. Age was treated as a continuous variable, while mobility decline and self-reported history of falls were coded into categorical variables. Disease severity as measured by the GMFCS-E&R was also classified as mild (Levels I and II) and moderate severity (Level III). An initial evaluation of the assumptions of the regression analyses led to a powered transformation of the dependent variables (PWI, MCS and PCS) in order to reduce skewness and the number of outliers, as well as to improve the normality of the residuals. All analyses were conducted using SPSS v20.0 (SPSS Inc, Chicago, IL).

## Results

### Participant characteristics

Thirty-four ambulant adults with CP participated ranging in age from 26 to 65 years (mean 44.2; SD 8.6), with the majority being female (*n* = 19). The predominant subtypes of spastic diplegia and hemiplegia were equally represented (*n* = 10 in each group). Four participants each described their CP subtype as either athetoid or mixed, and six people did not know or report their movement subtype. Five participants were at GMFCS-E&R Level I, fourteen were at Level II and fifteen were at Level III.

Almost all participants (n = 28; 82%) reported the occurrence of mobility decline since age 18. Similarly, almost every participant (33 out of 34) reported sustaining at least one fall in the past 12 months, with over 80% (n = 28) reporting more than two falls. Reported falls varied widely across participants with a median of 5 falls (IQR 9.0) and a maximum of 200.

### Health status

The health status of the cohort is reported in Table 1, including the MCS, PCS and the eight component dimensions of the SF-36. The mean PCS score for this sample was 50.3 (SD 10.7; 95% CI 46.5-54.0), which was similar to normative Australian data. The mean MCS score (mean 37.3; SD 12.8; 95% CI 32.9-41.8), however, was significantly lower than the overall population mean (mean 50.1; SD 10.0; 95% CI 50.0-50.2) as the 95% CI did not include the population mean [37]. Health perceptions of the dimensions varied, with highest mean ratings recorded for the role limitations (emotional) dimension and lowest for the physical functioning dimension. A Friedman test comparing the ratings between the eight dimensions of health status revealed significant differences (*p* < 0.0005). Post-hoc comparisons using the Wilcoxon Signed Rank tests with a Bonferonni adjustment showed that the ratings for vitality, physical function and general health were significantly lower compared to all other dimensions (*p* < 0.002). Of note, the rating for pain was also significantly lower compared to the rating for role limitations – emotional (*p* < 0.002).

**Table 1:** **HRQOL of ambulant adults with CP; data from the SF-36 and PWI (** ***n*** **= 34)**

**Domains**	**Mean (SD)**	**Median (IQR)**	**Range**	**95% CI**
SF-36				
Physical functioning	45.9 (30.2)	43 (56)	0-95	35.5, 56.4
Role limitations (physical)	69.9 (37.3)	88 (75)	0-100	56.8, 82.9
Role limitations (emotional)	87.3 (28.5)	100 (0)	0-100	77.3, 97.2
Vitality	51.0 (21.2)	58 (35)	0-80	43.6, 58.4
Emotional wellbeing	74.8 (13.7)	76 (14)	32-92	70.0, 79.6
Social functioning	71.8 (26.5)	75 (42)	13-100	62.6, 81.1
Bodily pain	58.8 (26.0)	56 (45)	0-100	49.7, 67.9
General health	56.0 (42.2)	50 (35)	10-100	47.6, 64.5
MCS	37.3 (12.8)	38 (22)	13-61	32.9, 41.8
PCS	50.3 (10.7)	52 (6)	13-69	46.5, 54.0
PWI				
Standard of living	66.2 (14.9)	70 (20)	10-90	59.6, 72.0
Personal health	53.8 (25.2)	55 (40)	0-90	43.9, 61.5
Achievements in life	64.1 (20.3)	70 (20)	10-90	56.4, 70.9
Personal relationships	70.6 (15.8)	70 (20)	20-90	64.7, 75.9
Personal safety	69.4 (19.8)	70 (23)	10-100	62.6, 76.8
Community connectedness	62.6 (22.5)	70 (33)	10-100	56.3, 71.6
Future security	64.8 (18.7)	70 (25)	10-90	58.2, 71.5
Summary index	65.2 (14.9)	69 (22)	21-89	59.8, 70.5

SF-36, Short Form-36 Health Survey; MCS, Mental Component Score; PCS, Physical Component Score; PWI, Personal Wellbeing Index; CI, confidence interval.

**Figure 1:**
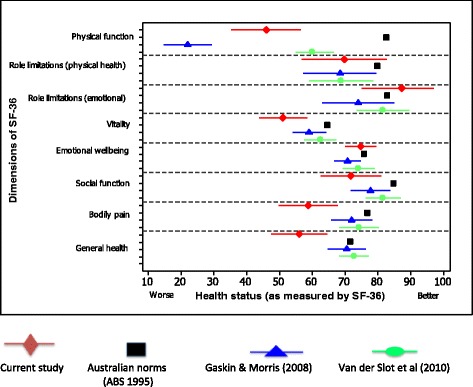
**Mean and 95% confidence intervals of health status as measured by the SF-36 for ambulant adults with CP (in red), the Australian general population (in black), all adults with CP (in blue) and Dutch adults with CP (in green).**

In addition to normative Australian data, the health status of this cohort was compared to that of Van der Slot [38] and Gaskin [22]. These are currently the only comparable data pertaining to adults with CP as both studies report scores from the 8 dimensions of the SF-36 as well as the PCS and MCS scores (Figure 1). Inspection of the CI suggests that there may be a difference in SF-36 ratings between ambulant adults with CP compared to those of an Australian normative group [37], with the exception of emotional wellbeing, role limitations–physical and role limitations–emotional. Health status ratings of this ambulant cohort also appeared to differ from a mixed mobility group of Australian adults with CP (*n* = 51) [22] in some dimensions, with the ambulant cohort reporting better physical function but poorer general health. The only difference detected in health status between our sample and predominantly ambulant adults with CP from the Netherlands (*n* = 56) [38] was in general health and bodily pain. Remaining dimensions demonstrated overlapping 95% confidence intervals around the estimates of these two samples.

### Well being

Data for the spirituality-religion domain of the PWI was not reported due to a low response rate (<30%) for this item. As shown in Table 1, the mean summary wellbeing (PWI-SI) score for this cohort was 65.2 (SD 14.9; 95% CI 59.8-70.5). This was significantly lower than the overall population mean (mean 75.0, SD 12.2; 95% CI 74.9-75.2) as the 95% CI for this sample did not include the population mean [36]. Participants reported that they were most satisfied with their personal relationships, personal safety and standard of living. Results of the Friedman test indicated that there was a significant difference in ratings across the seven PWI domains (*p* < 0.0005). Post-hoc comparisons using the Wilcoxon Signed Rank Test with a Bonferonni adjustment revealed that the rating for personal health was significantly lower compared to the ratings for standard of living, personal relationships and personal safety (*p* < 0.0024).

**Figure 2:**
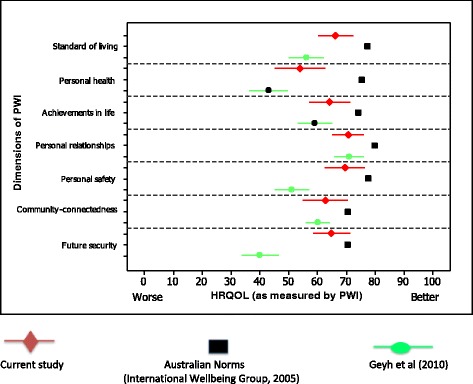
**Mean and 95% confidence intervals of wellbeing as measured by the PWI for the Australian general population (in black), Australian adults with CP (in red) and Australians with SCI (in green).**

In addition to normative Australian data, the PWI scores of a subset of Australian adults with acquired spinal cord injury (mean age 41 years, 44% incomplete lesion, 46% paraplegia, median 7 years post lesion) is provided for comparison [39]. To date, this is the only study that has reported PWI scores for a neurological population. Figure 2 illustrates the differences in PWI ratings for this cohort with CP compared to a normative Australian population [36] and a cohort with acquired spinal cord injury (SCI) (*n* = 40) [39].

### Relationship between HRQOL with mobility decline and falls history

A preliminary examination of potential differences in health status and well being between individuals who perceived a change in their mobility was explored using Mann–Whitney U tests with Bonferonni adjustment for multiple comparisons. As shown in Table 2, individuals with a decline in their mobility reported worse general health (*p* = 0.003) and more bodily pain (*p* = 0.002) compared to individuals whose mobility had not changed after entering adulthood. These individuals also experienced poorer satisfaction with their personal health (*p* = 0.004). No other significant differences were observed in the health status and well being ratings between the two groups.

**Table 2:** **Mann–Whitney results and group median differences in health status and well being for individuals who reported a decline in their mobility and have frequent falls**

	**Mobility declined**	**Mobility unchanged**	***p*** **-value** ^**1**^	**Frequent falls**	**No/Infrequent falls**	***p*** **-value** ^**2**^
SF-36						
Physical functioning	40.0	75.0	0.067	52.5	22.5	0.174
Role limitation (physical)	75.0	100.0	0.055	87.5	62.5	0.466
Role limitation (emotional)	100.0	100.0	0.725	100.0	50.0	**0.002**
Vitality	50.0	65.0	0.020	60.0	25.0	0.255
Emotional wellbeing	76.0	84.0	0.126	80.0	58.0	0.034
Social functioning	75.0	77.5	0.383	75.0	56.3	0.335
Bodily pain	55.0	93.8	**0.002**	56.3	61.3	0.909
General health	47.5	82.5	**0.003**	50.0	50.0	0.650
PWI						
Standard of living	70.0	75.0	0.547	70.0	50.0	0.043
Personal health	45.0	80.0	**0.004**	60.0	25.0	0.294
Achievements in life	70.0	70.0	0.645	70.0	65.0	0.310
Personal relationships	75.0	70.0	0.798	80.0	70.0	0.186
Personal safety	70.0	80.0	0.130	75.0	40.0	0.012
Community connectedness	5.0	70.0	0.552	70.0	60.0	0.208
Future security	70.0	70.0	0.569	70.0	60.0	0.191

SF-36, Short Form-36 Health Survey; PWI, Personal Wellbeing Index.

^1^ *p* < 0.006 (Mann–Whitney U Test with Bonferonni adjustment for multiple comparisons).

^2^ *p* < 0.007 (Mann–Whitney U Test with Bonferonni adjustment for multiple comparisons).

Significant results in bold.

Preliminary Mann–Whitney U tests showed that individuals who were non-or infrequent fallers perceived their health status to be worse in the SF-36 dimension of role limitations – emotional compared to frequent fallers (*p* = 0.002) (Table 2). No other statistically significant differences in SF-36 ratings were observed between the two groups. Similarly, there were no differences relating to falls history and the domains of the PWI.

### Predictors of HRQOL

As shown in Table 3, age, disease severity (GMFCS-E&R) and mobility decline were found by univariate regression analyses to be associated with mental health status as measured by the SF-36 MCS. In the multivariate model, however, only disease severity appeared to be a significant contributing factor (β = −0.47; *p* = 0.003). This model accounted for 48% of the variance in mental health status. For physical health status as measured by the SF-36 PCS, both the univariate and multivariate regression analyses showed that self-reported history of falls was the most important predictive factor (Table 3). The final model accounted for 21% of the variance in physical health status, with self-reported history of falls explaining 29% of the variance in SF-36 PCS scores. Disease severity and frequency of falls both significantly contributed to the variance in well being as measured by the PWI-SI in both univariate and multivariate regression analyses, although disease severity had a smaller contribution (13%) when other predictor variables were included (Table 3). The final model accounted for 35% of the variance in wellbeing. The most important predictive factor was self-reported history of falls, which accounted for 18% of the variance in PWI-SI scores.

**Table 3:** **Univariate and multivariate regression analyses of factors associated with aspects of HRQOL as measured by the SF-36 MCS, SF-36 PCS and PWI**

	**Mental health status (SF-36 MCS)**						**Physical health status (SF-36 PCS)**						**Life satisfaction (PWI)**					
	**Univariate**			**Multivariate** ^**1**^			**Univariate**			**Multivariate** ^**1**^			**Univariate**			**Multivariate** ^**1**^		
	β	95% CI	R^2^	β	95% CI	R^2^	β	95% CI	R^2^	β	95% CI	R^2^	β	95% CI	R^2^	β	95% CI	R^2^
Age	**0.5**	0.00, 0.01	20	0.2	−0.00, 0.01	3	0.0	−0.03, 0.03	0	−0.0	−0.03, 0.03	0	−0.1	−0.01, 0.01	0	−0.3	−0.01, 0.00	6
Disease severity	**−0.7**	−0.16, −0.07	42	**−0.5**	−0.12, −0.03	17	−0.1	−0.71, 0.30	2	−0.1	−0.58, 0.44	0	**−0.4**	−0.26, −0.04	20	**−0.4**	−0.25, −0.03	13
Mobility decline	**0.5**	0.05, 0.19	27	0.3	−0.01, 0.13	6	0.1	−0.51, 0.81	1	0.2	−0.35, 1.01	3	0.2	−0.05, 0.26	6	0.3	−0.04, 0.26	5
Falls	−0.0	−0.09, 0.07	0	−0.1	−0.08, 0.05	1	**−0.5**	−1.52, −0.38	27	**−0.6**	−1.61, −0.41	29	**−0.4**	−0.33, −0.04	17	**−0.4**	−0.32, −0.06	18

R^2^, unique contribution of each predictor variable to the variance in HRQOL in %.

^1^Enter method.

Significant results **(** ***p*** **< 0.05)** in bold.

## Discussion

This preliminary study described HRQOL in terms of wellbeing and health status and explored the relationship between falls and mobility decline with these self-report measures in a sample of ambulant Australian adults with CP. The proportion of individuals reporting mobility decline (>80%) and one or more falls in the previous year (>95%) was markedly higher in this sample compared to that reported in the literature, with 25-40% of adults with CP typically describing mobility decline [7,8], and 40-70% of ambulant adults with CP reporting falls [11,12,40]. It is likely that participants self-selected to take part in this study according to existing concerns regarding mobility and falls, resulting in the increased proportion observed.

Wellbeing as measured by the PWI-SI and most individual PWI domains were below that of the general Australian population [37], with least satisfaction with personal health. Similarly, Australian ambulant adults with CP appear to have poorer health status in the areas of physical function, vitality, social function, bodily pain and general health compared to the Australian general population, as measured with the SF-36. Van der Slot and colleagues similarly demonstrated lower health status in a Dutch cohort of ambulant adults with CP compared to Dutch normative data [38]. This and our findings accords with previous findings that adults with CP experience life as less manageable and meaningful, and above all ‘more unpredictable and incomprehensible’ than the general population [41], impacting on well being and health status. More detailed direct comparisons of wellbeing and health status with other cohorts of adults with CP is limited due to different measurement instruments, variation in intellectual and mobility capacity of participants, and cultural influences [17,19,42]. However, accumulating research to date suggests that older age [17], limited physical activity [22], fatigue [21], pain [18,19], and visual loss [6], may be negatively associated with the physical and/or mental aspects of HRQOL in adults with CP.

In contrast, adults with CP in this study rated their wellbeing (as measured by PWI-SI) higher than those with adult onset spinal cord injury [39]. Absence of illness or disability is not a pre requisite for health; therefore individuals living with a developmental disability can consider themselves generally ‘healthy’ [43]. It is possible that those ageing with CP having lived with disability all their lives have slightly different viewpoints on ‘health’ and life expectations to those with a more recently acquired disability, such as spinal cord injury. The variation in wellbeing for those with developmental versus acquired disability warrants further exploration.

Mobility decline may be associated with poorer satisfaction with personal health (PWI) and lower ratings of general health (SF-36). A greater understanding of factors associated with mobility decline, such as age of walking debut, use of gait aids during childhood (GMFCS Level III), and the presence of bilateral movement dysfunction has arisen over the past 10 or so years [7]. Not surprisingly, the findings of this preliminary study supports previous research revealing that mobility decline also impacts aspects of HRQOL, potentially through mechanisms such as functional loss, pain, fatigue, and reduction in participation opportunities [18,19]. It may be beneficial for adult disability services to provide health promotion strategies including education around ageing to assist ambulant adults with CP navigate adulthood where pre-existing mobility skills, may be lost or reduced, with the potential for lowered HRQOL.

In this small sample, people who did not fall or fell infrequently reported lower ‘role limitation–emotional’ (SF-36) than those who fell often. This dimension explored the impact of emotional problems (such as depression or anxiety) on an individual’s ability to complete work or other duties, accomplish tasks and be careful in work and other tasks. A low score in this domain suggests that a person’s daily activity is limited by emotional problems (fear, anxiety, depression). It may be that those who do not fall or fall only infrequently have considerable fear or anxiety regarding their mobility, with the need to implement constant vigilance to remain falls-free, subsequently restricting social and participation opportunities. Restriction of activity in those at high risk of falls has been documented in other populations [24,25]. It is possible that those who experience falls, also experience relatively greater social and participation opportunities, albeit whilst putting themselves at risk. Whilst this finding needs to be interpreted with caution, the relationship between falling behaviour and emotional health in adults ageing with CP warrants further research.

Falls history was found to be a significant contributor to the model developed for physical health status (SF-36 PCS), accounting for 29% of the variance, and 18% of the variance in wellbeing (PWI-SI scores) in this small sample of ambulant adults with CP. Unlike other factors considered in this study such as GMFCS level which is unlikely to improve over adulthood [44], or age which cannot be altered, there may be the potential to enhance HRQOL by addressing falls behaviours in ambulant adults with CP. Further prospective studies are therefore required in order to examine the relationship between ambulant adults with CP who do or do not fall and their HRQOL.

One of the main limitations of this study was the small sample of participants who reported that their mobility did not decline and who did not have a history of falls. In addition, this study is likely to have particularly appealed to participants already concerned about mobility decline and falls. The falls rate and profile may therefore have been overestimated compared to that of the wider population of ambulant adults with CP, and caution is warranted when interpreting the results. In order to validate the findings of this study, further studies with significantly larger sample sizes are required. Furthermore, the findings can only be applied to adults with sufficient cognition to complete detailed surveys. However, given that cognitive impairment is more typically experienced by those at GMFCS Levels IV– V [45], reasonable inferences can be made regarding the findings to the subset of ambulant adults with CP. Despite these limitations, useful preliminary data has been gathered describing experiences of ambulant adults with CP regarding well being and health status, and the impact of mobility decline and falls on aspects of HRQOL.

## Conclusion

This preliminary study has demonstrated that ambulant adults with CP may report wellbeing and health status domains below that of age- and sex-matched normative Australian data, particularly those related to physical health. It has also shown that physical health status and personal wellbeing might be negatively associated with a history of falls. Further prospective research is needed in order to better understand the relationship between HRQOL, falls and mobility decline. This includes examining the efficacy of falls prevention programs on life quality in this population.

## Consent

Written informed consent provided by participants for this report to be published.
